# The Structural and Magnetic Properties of Fe^II^ and Co^II^ Complexes with 2-(furan-2-yl)-5-pyridin-2-yl-1,3,4-oxadiazole

**DOI:** 10.3390/molecules25020277

**Published:** 2020-01-09

**Authors:** Pavel Zoufalý, Erik Čižmár, Juraj Kuchár, Radovan Herchel

**Affiliations:** 1 Department of Inorganic Chemistry, Faculty of Science, Palacký University, 17. Listopadu 12, CZ-771 46 Olomouc, Czech Republic; pavel.zoufaly@upol.cz (P.Z.); or; 2 Institute of Physics, Faculty of Science, P.J. Šafárik University in Košice, Park Angelinum 9, SK-041 54 Košice, Slovakia; erik.cizmar@upjs.sk; 3 Department of Inorganic Chemistry, Institute of Chemistry, Faculty of Science, P.J. Šafárik University in Košice, Moyzesova 11, SK-041 54 Košice, Slovakia

**Keywords:** 1,3,4-oxadiazole, single-molecule magnet, second coordination sphere

## Abstract

Two novel coordination compounds containing heterocyclic bidentate *N*,*N*-donor ligand 2-(furan-2-yl)-5-(pyridin-2-yl)-1,3,4-oxadiazole (fpo) were synthesized. A general formula for compounds originating from perchlorates of iron, cobalt, and fpo can be written as: [M(fpo)_2_(H_2_O)_2_](ClO_4_)_2_ (M = Fe(II) for (**1**) Co(II) for (**2**)). The characterization of compounds was performed by general physico-chemical methods—elemental analysis (EA), Fourier transform infrared spectroscopy (FT-IR), nuclear magnetic resonance (NMR) in case of organics, and single crystal X-ray diffraction (sXRD). Moreover, magneto-chemical properties were studied employing measurements in static field (DC) for **1** and X-band EPR (Electron paramagnetic resonance), direct current (DC), and alternating current (AC) magnetic measurements in case of **2**. The analysis of DC magnetic properties revealed a high spin arrangement in **1**, significant rhombicity for both complexes, and large magnetic anisotropy in **2** (*D* = −21.2 cm^−1^). Moreover, **2** showed field-induced slow relaxation of the magnetization (*U*_eff_ = 65.3 K). EPR spectroscopy and ab initio calculations (CASSCF/NEVPT2) confirmed the presence of easy axis anisotropy and the importance of the second coordination sphere.

## 1. Introduction

**Scheme 1:**
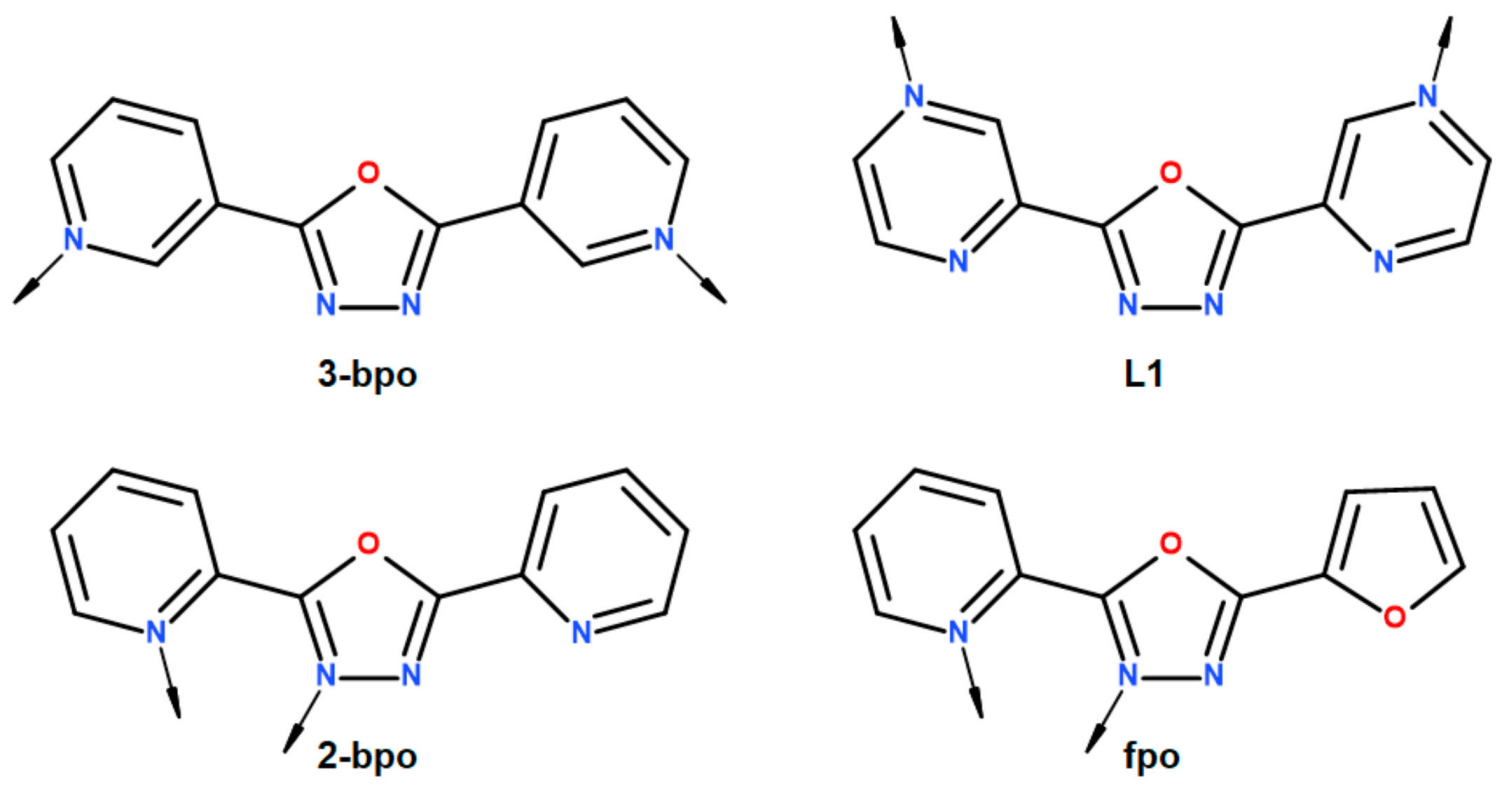
Structural formulas of studied ligand fpo and ligands discussed in the text comprising 1,3,4-oxadiazole moiety. Various coordination modes are depicted with arrows.

The main focus of magnetochemical research is situated in the study of molecular functional materials exhibiting bistability in which physical properties can be triggered by a change of temperature, pressure, light or magnetic field, such as spin crossover compounds (SCO) or single-molecule magnets (SMM), where the latter are nanomagnets characterized by the slow relaxation of the magnetization. The widespread basis for the study of these phenomena originates in its potential technological applications in many areas of everyday life. In this case, SMM compounds are studied as materials for storage devices, quantum computing, sensors, and spintronics [1,2]. Additionally, SCO compounds may find potential in many applications such as pressure or optical switches [3], gas sensors [4], pressure, or temperature sensors [5]. Therefore, the magnetic properties of 3d elements are of much interest to the scientific community. Among all of the 3d metals, Co(II) possesses great predispositions for the formation of compounds exhibiting SMM behavior due to relatively large spin-orbit coupling and the possibility to vary the coordination numbers from 2 to 8 [6]. Likewise, Fe(II) ranks among elements whose compounds are commonly known for SCO behavior [7]. The tuning of physico-chemical properties of both SMM and SCO phenomena can be achieved by means of several factors. In SMMs, complexes honing of magnetization reversal barrier quantified by *U*_eff_ parameter and magnetic blocking temperature (*T*_B_) is achieved by the strengthening of magnetic anisotropy of the easy-axis type. The modification of *U*_eff_ is accomplished by increasing magnitude of the zero-field splitting parameter *D*, due to a simple relationship among them, *U* = |*D*|(S^2^ − 1/4) for half-integer spin and *U* = |*D*|(S^2^) for integer spin, and it holds that values of ZFS (zero-field splitting) parameters are dictated by the coordination number, the ligand field, and the symmetry of the coordination polyhedron. In general, higher |*D|*-values should yield higher *U*_eff_ and correspondingly higher *T*_B_ [8,9,10,11]. However, large zero-field splitting parameter *E* causes an increase of the tunneling rate of the magnetization. In SCO compounds, a proper ligand field and cooperativity between neighboring molecules can allow a transition between low spin (LS) and high spin (HS) state and vice versa, which is triggered by the change of temperature or pressure, eventually by the light. Since many of these parameters in both SMM and SCO relate to the coordination environment, search for suitable organic ligands is crucial for improving properties of these molecular compound classes. Miscellaneous organic ligands have been employed to study or enhance the magnetic properties of Fe(II) and Co(II) coordination compounds. Such ligands frequently include five or six-membered heterocycles containing at least one nitrogen atom. Examples of these heterocycles cover substituted diazoles, triazoles, tetrazoles, pyridines, pyrimidines, pyrazines, triazines, and others [12,13,14]. In our search for suitable ligands, we were inspired by numerous publications with interesting magnetochemical results encompassing substituted triazoles, and more specifically 4-amino-3,5-di-2-pyridyl-4*H*-1,2,4-triazole (abpt). Our co-workers participated in publishing Co(II) field-induced single-ion magnets incorporating the abpt ligand. The first publication in 2014 introduced [Co(abpt)_2_(tcm)_2_] (tcm = tricyanomethanide), which was identified as the field-induced single-ion magnet with large ZFS parameters and positive *D* = 48 cm^−1^. The energy value for spin reversal barrier *U*_eff_ = 86.2 K was the highest at the time among Co(II) complexes with transversal magnetic anisotropy [15]. The research continued in series of two publications involving other pseudohalide analogs—[Co(abpt)_2_(solv)_2_]X_2_ (solv = H_2_O and X = tcap, solv = H_2_O and X = nodcm, solv = CH_3_OH and X = pcp) (tcap = 1,1,3,3-tetracyano-2-azapropenide, nodcm = nitrodicyanomethanide, pcp = 1,1,2,3,3-pentacyanopropenide), [Co(abpt)_2_(X)_2_] (X = nca, NCSe, ndcm, N_3_) (nca = nitrocyanamide, ndcm = nitroso-dicyanomethanide). The analysis of DC measurements revealed strong magnetic anisotropy and *D* in 31–41.4 cm^−1^ range with the exception of [Co(abpt)_2_(N_3_)_2_] where *D* = −24.1cm^−1^. Subsequent inquiry of AC data showed field-induced slow relaxation of the magnetization and *U*_eff_ in the scope of 71.6–108 cm^−1^ [16,17]. Thus, we have decided to explore 1,3,4-oxadiazoles, oxygen analogs of abpt, which are relatively unexplored in a magnetochemical area. Moreover, the fact that 4*H*-1,2,4-triazole analog 1,3,4-oxadiazole contains more electron-withdrawing oxygen instead of nitrogen should have a notable effect on magnetic properties. Generally, studies involving a 1,3,4-oxadiazole heterocycle focus on crystal engineering, which covers the design and preparation of building blocks, distinguished crystal structures, and understanding of intermolecular interactions in supramolecular structure. Moreover, in these studies, nitrogen of the 1,3,4-oxadiazole ring do not coordinate, and, instead, substituents (e.g., pyridine, pyrazine) in position 2 or 5 provide suitable donor atoms (Scheme 1). The structures of [Co(3-bpo)(dca)_2_]_n_ (dca = dicyanamide, 3-bpo = 2,5-bis(3-pyridyl)-1,3,4-oxadiazole) and [Co(L1)_2_(SCN)_2_(H_2_O)_2_](L1)_2_(H_2_O)_6_ (L1 = 2,5-bis(2-pyrazinyl)-1,3,4-oxadiazole) may serve as a good example [18,19]. Studies comprising of Co(II) complexes with 1,3,4-oxadiazole derivatives in which cobalt atom is directly bound to a nitrogen atom of 1,3,4-oxadiazole were focused mainly on biological activities of these compounds, and, sporadically, DC magnetic properties were also reported [20,21,22,23,24,25]. Likewise, a few coordination compounds of Fe(II) with 1,3,4-oxadiazole directly bonded through its nitrogen atom appear in literature, e.g., [Fe(2-bpo)_2_(H_2_O)_2_](ClO_4_)_2_ (2-bpo = 2,5-bis(2-pyridyl)-1,3,4-oxadiazole) was prepared and found to be in the high spin state in the 2-300 K range [26]. Magnetochemically focused study exploring Light-Induced Excited Spin-State Trapping (LIESST) and SCO in 2,5-bis(2-pyridyl)-1,3,4-chalcadiazoles describes magnetic properties of [Fe(2-bpo)_2_(NCS)_2_]. Despite the fact that the complex contains two NCS coordinated anions creating stronger ligand field, the occurrence of SCO was not observed in the 2–300 K temperature scale [27]. The very first SCO compounds based on 1,3,4-oxadiazole were synthesized and, subsequently, published in 2016 by C. Köhler and E. Rentschler in series of [Fe^II^_2_(μ-L)_2_]Y_4_·*x*CH_3_CN (L = 2,5-bis{[(2-pyridylmethyl)amino]-methyl}-1,3,4-oxadiazole; Y = ClO_4_^−^, BF_4_^−^ and CF_3_SO_3_^−^; *x* = 4 for ClO_4_^−^ and *x* = 2 for BF_4_^−^, CF_3_SO_3_^−^). The dinuclear complex with the BF_4_^–^ anion remained in the high-spin (HS) state in the 10–300 K range whereas, in the case of ClO_4_^−^ and CF_3_SO_3_^−^ anions, SCO was observed. The critical temperature *T*_1/2_ occurs somewhere around 150 K for both anions and, in the case of [Fe^II^_2_(μ-L)_2_](CF_3_SO_3_)_4_·2CH_3_CN, steep hysteresis of about 26 K accompanies the transition. XRD confirmed the [HS-HS] to [LS-HS] spin transition, where only one of the Fe(II) atoms undergoes SCO [28]. These complexes were also studied by theoretical methods (DFT and CASSCF/CASPT2) [29]. The above-mentioned studies show the great potential of 1,3,4-oxadiazole ligands in the molecular magnetism.

Herein, the 2-(furan-2-yl)-5-pyridin-2-yl-1,3,4-oxadiazole (fpo) was utilized in the preparation of [M(fpo)_2_(H_2_O)_2_](ClO_4_)_2_ (M = Fe(II) for (**1**) Co(II) for (**2**)) coordination compounds, which were characterized by single-crystal X-ray analysis, FT-IR spectroscopy, and EPR spectroscopy. Both static and dynamic magnetic properties of these complexes were investigated and the analysis was supported by DFT and CASSCF/NEVPT2 theoretical methods showing the importance of the second coordination sphere on the zero-field splitting parameters.

## 2. Results and Discussion

### 2.1. Synthesis

**Scheme 2:**
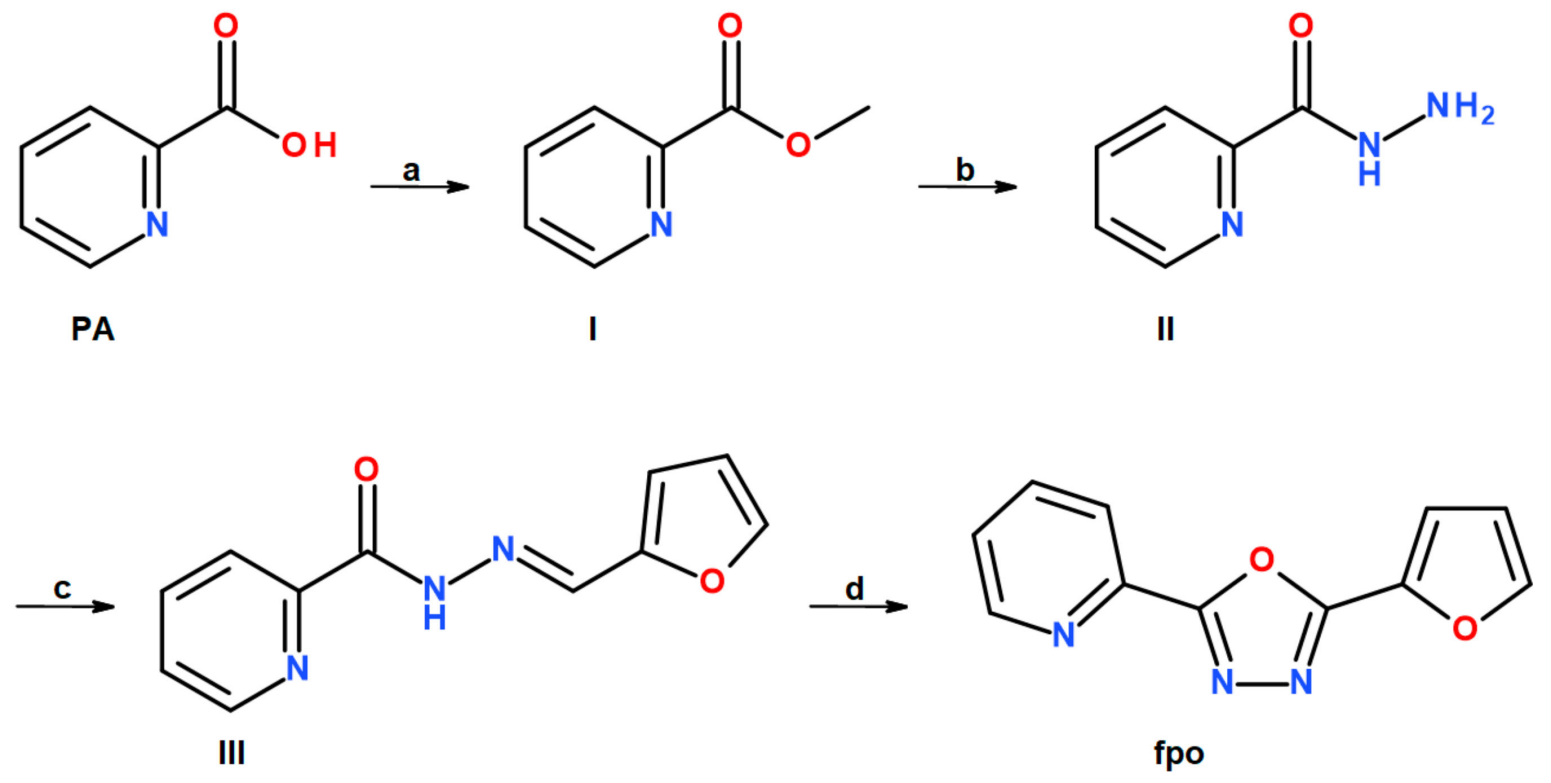
Preparation of the fpo ligand in four steps: (**a**) methanol, sulphuric acid, 24 h reflux, (**b**) methanol, hydrazine hydrate, 14 h reflux, (**c**) methanol, furfural, 2 h reflux, (**d**) iodine, potassium carbonate, 18 h, 120 °C.

Preparation of ligand 2-(furan-2-yl)-5-(pyridin-2-yl)-1,3,4-oxadiazole (fpo) is already described in the literature [30]. However, we obtained fpo through a four-step reaction, which shows simplified reaction Scheme 2. In the first step, transformation of picolinic acid (PA) to methyl picolinate (I) occurs via esterification in methanol/sulphuric acid solution. In the second step, production of picolinic acid hydrazide (II) from I ensues in methanol/hydrazine hydrate solution. The third step involves the conversion of II into picolinic acid 2-(2-furanylmethylene)hydrazide (III) in a simple reaction with 2-furaldehyde in methanol. The last step shows oxidative cyclization of the imine bond in dimethylsulfoxide with iodine as an oxidation reagent and potassium carbonate as the base. Subsequently, dissolving M(II) perchlorate hexahydrate (M = Fe, Co) in methanol and adding the solution to fpo in methanol and reflux of the mixture results in the formation of the products. Within one week, yellow crystals of **1** and orange crystals of **2** appeared. Since all organic ligands are already recorded in literature, their synthesis was inspired by these protocols [31,32,33]. To confirm the structure of I, II, III, and fpo, the measurements of IR and NMR spectra were employed.

### 2.2. Description of the Molecular and Crystal Structure

**Figure 1:**
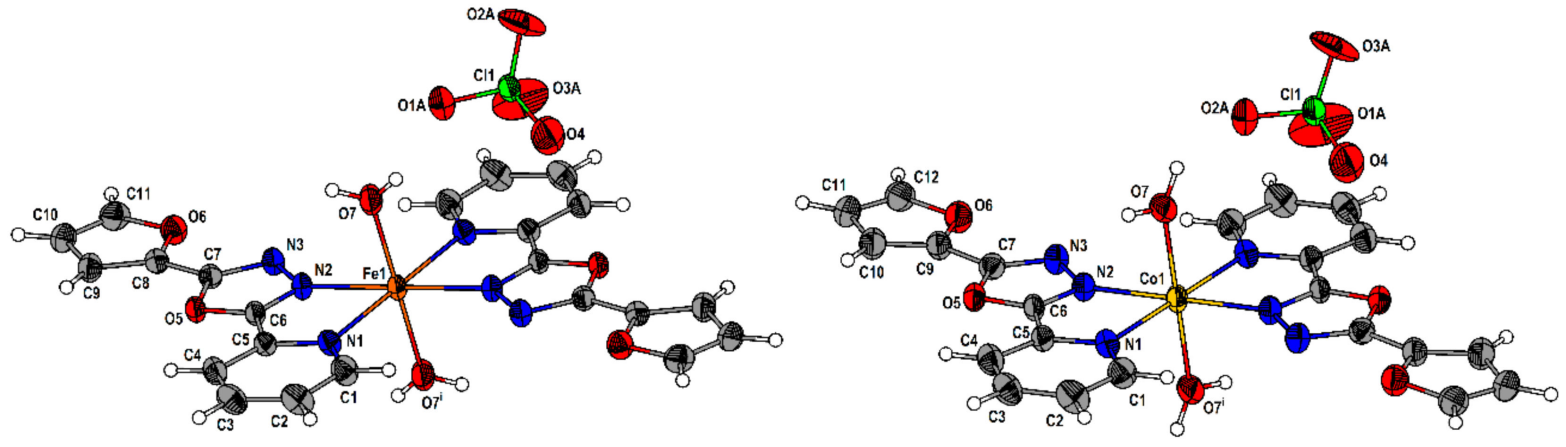
The molecular structures of [Fe(fpo)_2_(H_2_O)_2_](ClO_4_)_2_ (1, left) and [Co(fpo)_2_(H_2_O)_2_](ClO_4_)_2_ (2, right). The most populated part of the perchlorate anion is depicted. Symmetrical codes: i: 1-x, 1-y, 1-z.

Compounds [Fe(fpo)_2_(H_2_O)_2_](ClO_4_)_2_ (**1**) and [Co(fpo)_2_(H_2_O)_2_](ClO_4_)_2_ (**2**) are isostructural and crystallize in the monoclinic *P*2_1_/*c* space group. In both compounds, the central atoms occupy the center of the symmetry. Two molecules of the fpo ligand are bidentately coordinated to central atom via nitrogen (N_ox_ and N_py_), and two coordinated oxygens from water molecules are in the apical positions. This arrangement constitutes {FeN_4_O_2_} and {CoN_4_O_2_} chromophores. Therefore, both metal ions are situated in a typical octahedral coordination sphere where distortion occurs mainly due to the chelate binding of the fpo ligand. The relevant bite angle between pyridine nitrogen (N_py_), iron, and nitrogen of 1,3,4-oxadiazole (N_ox_) of 1 measures 75.94(9)° and 76.88(15)° in 2. Thus, the parameter for angular distortion *∑* calculated as the sum of deviation of 12 angles from the ideal 90° angle yields 61° for Fe(II) and 55.6° for Co(II). The bond length of Fe(II) and pyridine nitrogen is *d*(Fe-N_py_) = 2.212(3) Å, whereas lower value of *d*(Fe-N_ox_) = 2.165(2) Å appears in 1,3,4-oxadiazole nitrogen and lowest for aqua oxygen *d*(Fe-O) = 2.078(3) Å. Similar but shorter distances are found in the Co(II) complex *d*(Co-N_py_) = 2.159(4) Å, *d*(Co-N_ox_) = 2.116(4) Å, and *d*(Co-O) = 2.064(4) Å. The molecular structures of 1 and 2 are depicted in Figure 1.

**Figure 2:**
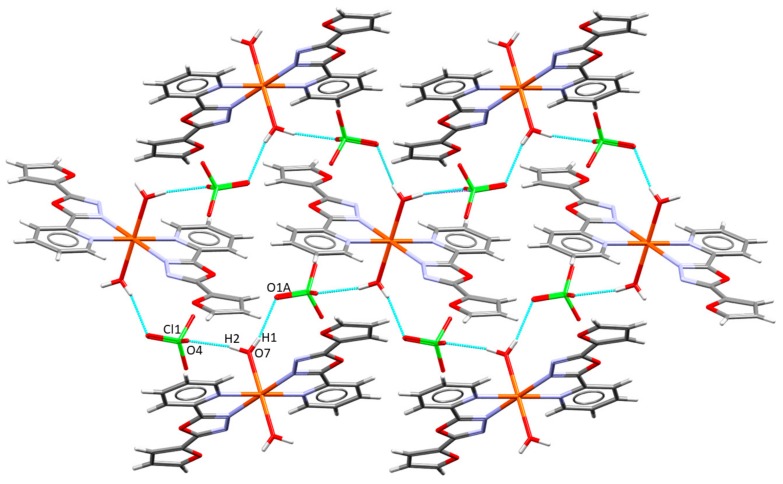
The supramolecular 2D network formed through hydrogen bonds among perchlorates and aqua ligands in the crystal structure of 1. Only atoms involved in the hydrogen bonds are labelled.

Apart from covalent bonds, noncovalent interactions create a rich net, which originates mainly from oxygen atoms of perchlorates and aqua ligands. Hydrogen bonds between hydrogen atoms of aqua and perchlorate oxygens form the most prominent structural element. Every hydrogen bond from aqua links with different perchlorate oxygen including four ClO_4_^−^ anions in total per [M(fpo)_2_(H_2_O)_2_]^2+^ unit. The shorter distance of the hydrogen bond between oxygens measures *d*(O···O) = 2.727(6) Å and longer 2.807(4) Å in 1, *d*(O···O) = 2.726(9) Å, and *d*(O···O) = 2.826(6) Å in 2. The perchlorate anion creates only two hydrogen bonds and each come with a different aqua ligand. Propagation of the hydrogen bonds forms a 2D layer as depicted in Figure 2**.** Basic crystallographic data and parameters are shown in Table 1.

### 2.3. Static and Dynamic Magnetic Properties

**Figure 3:**
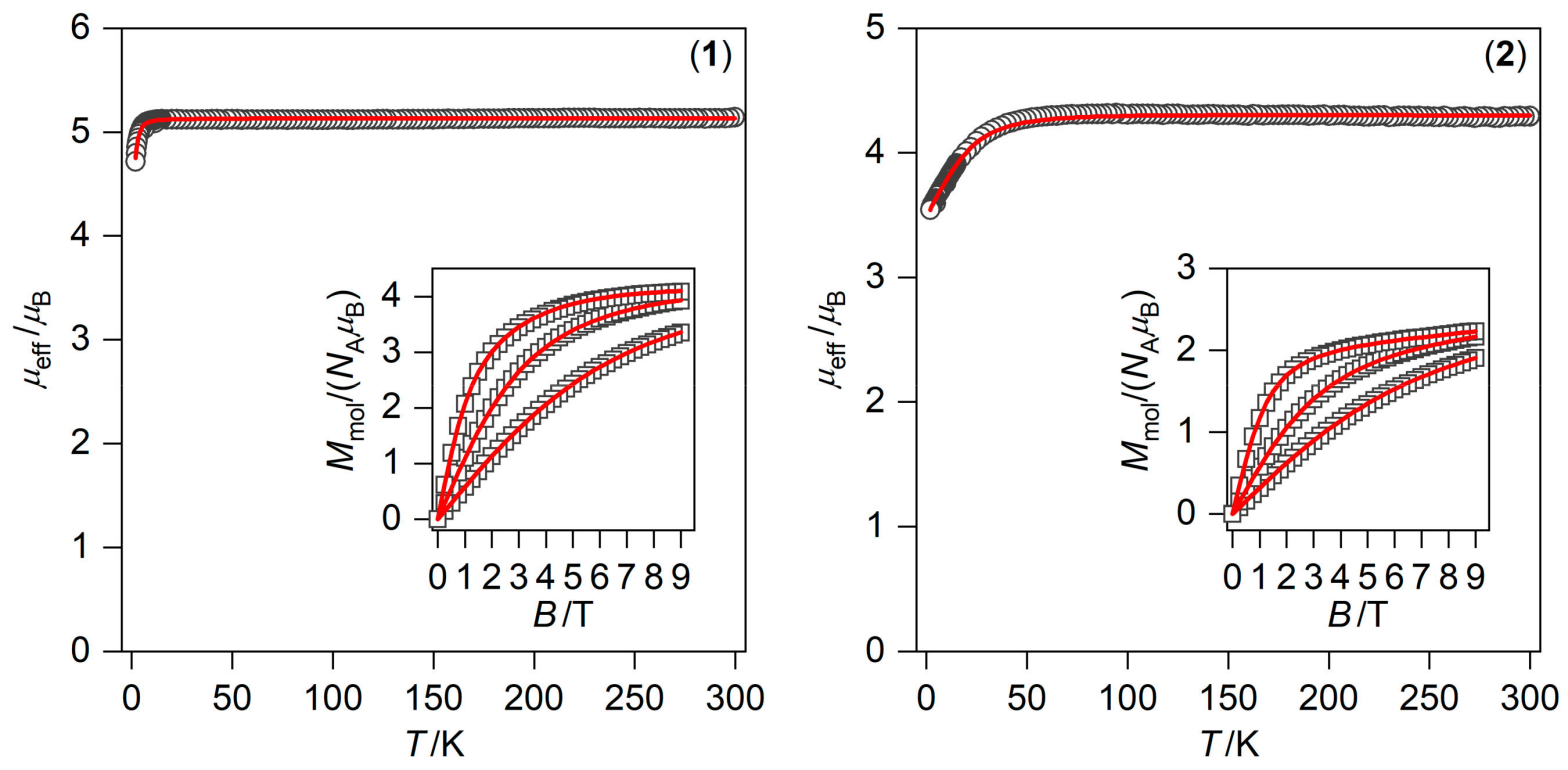
Magnetic data for 1 and 2 shown as the temperature dependence of the effective magnetic moment and as the isothermal magnetizations measured at *T* = 2, 5, and 10 K. The empty circles represent the experimental data. Red full lines represent the fitted data using Equation (1) with *D* = −1.23 cm^−1^, *E*/*D* = 0.31, *g*_iso_ = 2.09 for **1** and *D* = −21.2 cm^−1^, *E*/*D* = 0.32, *g*_xy_ = 2.13, *g*_z_ = 2.37 for 2.

The variable temperature and field experimental magnetic data for 1 and 2 are plotted in Figure 3. The room temperature effective magnetic moments (*μ*_eff_) adopt values 5.1 *μ*_B_ for 1 and 4.3 *μ*_B_ for 2. These values are a bit higher than the spin-only values for *S* = 2 (*μ*_eff_/*μ*_B_ = 4.9) and for *S* = 3/2 (*μ*_eff_/*μ*_B_ = 3.9) calculated for *g* = 2.0, which indicates a significant contribution of the spin-orbit coupling to the ground state. On lowering the temperature to 1.9 K, there is only a small decrease of the effective magnetic moment for 1 down to 4.7 *μ*_B_, whereas compound 1 exhibit a much larger drop of *μ*_eff_ down to 3.5 *μ*_B_. These data suggest that magnetic anisotropy in 2 is much more pronounced in comparison to 1. This is also reflected in the isothermal magnetization data measured at *T* = 2 K saturating to *M*_mol_/*N*_A_*μ*_B_ = 4.1 for 1 and to *M*_mol_/*N*_A_*μ*_B_ = 2.2 for 2, when compared to the theoretical limit values of *M*_mol_/*N*_A_*μ*_B_→*g***·***S ≈* 4 for 1 and 3 for 2. Moreover, there are no maxima of the molar susceptibility at low temperature for both compounds under study. Thus, we can exclude significant antiferromagnetic interactions in the solid state.

Therefore, the experimental magnetic data were analyzed with the following spin Hamiltonian suitable for describing magnetic anisotropy.

(1) $$\hat{H}=D\left({\hat{S}}_{z}^{2}-{\hat{S}}_{}^{2}/3\right)+E\left({\hat{S}}_{x}^{2}-{\hat{S}}_{y}^{2}\right)+\mu_{B}Bg{\hat{S}}_{a}^{}$$

where the single-ion ZFS term is described by axial *D* and rhombic *E* parameters, and the Zeeman term is defined in the α-direction of the magnetic field as *B_α_* = *B*(sin(*θ*)cos(*φ*), sin(*θ*)sin(*φ*), cos(*θ*)) [34]. The molar magnetization (*M_a_*) was then calculated from the partition function (*Z*) for a given direction of magnetic field *B_α_* as:

(2) $$M_{a}=N_{A}kT\frac{\partial \ln Z}{\partial B_{a}}$$

and the integral (orientational) average of the molar magnetization was calculated as:

(3) $$M_{\mathrm{mol}}=\frac{1}{4\pi }\int_{\varphi =0}^{2\varphi }\int_{\theta =0}^{\pi }M_{a}\sin\theta d\theta d\varphi$$

to properly simulate experimental powder magnetization data. To obtain trustworthy parameters, both temperature and field-dependent magnetic data were fitted simultaneously. Moreover, we have tested both possible signs of the *D*-parameter for both compounds 1 and 2. The value of |*D*|~1.2 cm^−1^ for 1 was found, which is rather small, but, in the case of compound 2, |*D*| is around 20 cm^−1^, which confirms large magnetic anisotropy. In addition, the significant rhombicity was observed in both compounds (*E*/*D* >> 0)—Table 2 and Table 3.

The large magnetic anisotropy in 2 encouraged us to measure AC susceptibility data for this compound. The zero-static magnetic field measurements result in a zero signal of the imaginary part of AC susceptibility. The application of small static DC field confirmed slow relaxation of the magnetization (Figure S1). Therefore, small static field (*B*_DC_ = 0.1 T) was chosen to suppress the tunneling of the magnetization and to avoid induction of the magnetic dipolar interactions in solid state, which often cause the appearance of another relaxation pathway. Thus, the temperature-dependent AC susceptibility data were acquired for the range of frequencies 1–1500 Hz, and these data were further analyzed with the one-component Debye model based on Equation (4).

(4) $$\chi \left(\varpi \right)=\frac{\chi_{T}-\chi_{S}}{1+{\left(i\varpi \tau \right)}^{1-\alpha }}+\chi_{S}$$

**Figure 4:**
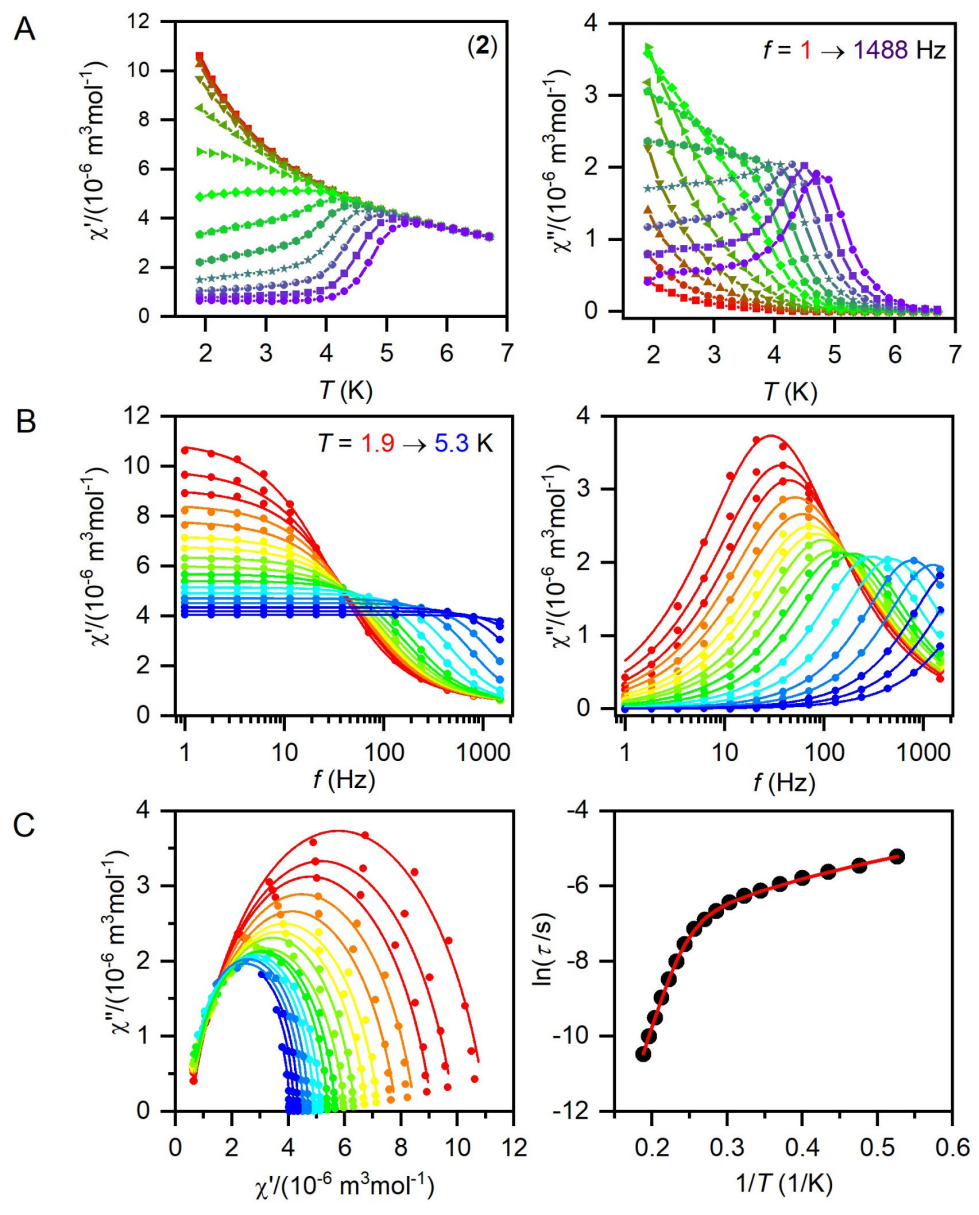
AC susceptibility data for 2. **A**: in-phase *χ*′ and out-of-phase *χ*′′ molar susceptibilities at the applied external magnetic field *B*_DC_ = 0.1 T (full lines are only guides for eyes). **B**: frequency dependence of in-phase *χ*′ and out-of-phase *χ*′′ molar susceptibilities fitted with one-component Debye’s model using Equation (4) (full lines). **C**: The Argand (Cole-Cole) plot with full lines fitted with Equation (4) and on the right of the fit of resulting relaxation times *τ* with the combination of Raman and Orbach processes (red line) using Equation (5).

This resulted in isothermal (*χ*_T_) and adiabatic (*χ*_S_) susceptibilities, relaxation times (*τ*), and distribution parameters (*α*) (Table S1), and the Argand (Cole-Cole) plot (Figure 4). Then, the temperature dependence of the relaxation times was fitted to the combination of two-phonon Raman and Orbach relaxation processes, which is shown below.

(5) $$\frac{1}{\tau }=CT^{n}+\frac{1}{\tau_{0}}\exp\left(-\frac{U_{\mathrm{eff}}}{kT}\right)$$

This results in *C* = 44.8 K^−n^s^−1^, *n* = 2.19, *τ*_0_ = 1.34 × 10^−10^ s^−1^, and *U*_eff_ = 65.3 K (Figure 4). It is worth mentioning that the other combinations of the relaxation processes were tested, like direct and Raman, or direct and Orbach, but these were unsuccessful. The found spin reversal barrier *U*_eff_ = 65.3 K = 45.4 cm^−1^ is in good agreement with a value of 48.3 cm^−1^ derived with fitted parameters from DC magnetic data (*D* = −21.2 cm^−1^, *E*/*D* = 0.32). The Raman parameter *n* is shifted to lower values than the theoretical value of 9, which is usually ascribed to the involvement of both acoustic and optical phonons in the relaxation [35].

### 2.4. Electron Paramagnetic Spectroscopy

**Figure 5:**
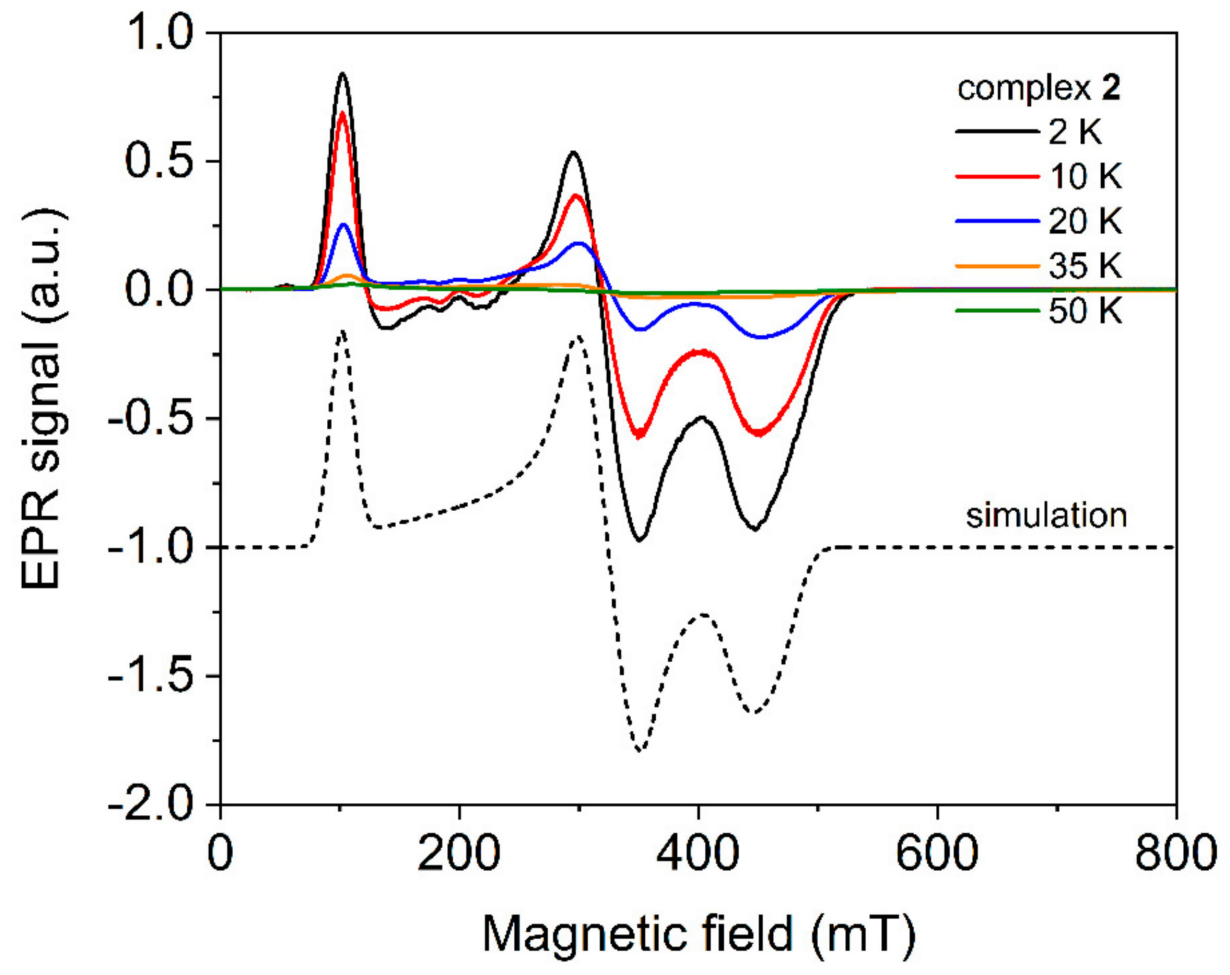
Temperature evolution of the EPR spectra of 2 measured with the excitation frequency of 9.4 GHz (solid lines). The simulation using the effective spin *S*_eff_ = 1/2 model (black dashed line) with the parameters described in the text is included.

The X-band spectra of electron paramagnetic resonance (EPR) of complex 2 were measured in the temperature range from 2 K to 70 K to identify the type of the crystal-field anisotropy. The temperature evolution of the EPR spectra of complex 2 (Figure 5) shows a typical decrease of the signal intensity with increasing temperature for Co(II) complexes. Since there is no change of the shape of the spectra in the whole temperature range, a simplified effective spin *S*_eff_ = 1/2 model due to the expected strong splitting between the ground and excited Kramers doublets was used for the analysis. This model assumes the mixing of higher excited states with the ground Kramers doublet as the consequence of the spin-orbit coupling, which yields highly anisotropic effective *g*-factors. The simulation of EPR spectra was performed within the EasySpin simulation package [36]. The influence of the unresolved hyperfine coupling was included at first only by an anisotropic convolutional broadening. To fairly reproduce the experimental data, the anisotropic hyperfine interaction term *A* was then included together with an anisotropic convolutional broadening Δ*B* (full-width at half-height). The best agreement with the experiment was obtained using the set *g*_1_ = 1.48, *g*_2_ = 2.06, *g*_3_ = 6.60, *A*_1_ = 140 MHz, *A*_2_ = 165 MHz, *A*_3_ = 160 MHz, Δ*B*_11_ = 22 mT, Δ*B*_12_ = 30 mT, and Δ*B*_13_ = 7 mT. It should be noted that the spin-Hamiltonian formalism does not allow us to estimate the value of the *D* parameter for large values (only *E*/*D* ratio). The fitted g-factors are in good agreement with the parameters derived from ab initio calculations in Table S2 (*vide infra*). Our attempts to simulate the EPR spectra using the spin-Hamiltonian formalism yielded the spectra similar to the experimental ones for both positive and negative values of the *D*-parameter with *E*/*D* ranging from 0.305 to 0.330. The values of the effective g-factors from the analysis of the EPR spectra using the effective spin *S*_eff_ = 1/2 model allow revealing the type of the anisotropy (easy-axis or easy-plane) when compared with their theoretical prediction using Griffith-Figgis formalism or with ab initio calculations [37]. Within the Griffith-Figgis formalism (with axial Δ_ax_ and rhombic Δ_rh_ crystal field term included), the calculated effective g-factors components, using a similar approach as in Reference [38], are restricted to *g* ≥ 2 for the positive axial field (easy-plane anisotropy). A very high value of *g*_3_ = 6.60 is very close to the predicted *g*_z_ component in the case of a negative axial field (easy-axis anisotropy) higher than 1500 cm^−1^. In that case, the obtained *g*_1_, *g*_2_, and *g*_3_ components of the effective g-factor can be assigned as *g*_x_ = 2.06, *g*_y_ = 1.48, and *g*_z_ = 6.6. Although, in that case, the predicted difference between the ground and the first excited Kramers doublet Δ_1–2_ is approximately 150 cm^−1^. The study of Titiš et al. shows that an additional deformation of the coordination octahedron, e.g., so-called scissoring as present in 2, will yield to the reduction of Δ_1–2_ [39]. The experimental estimation of the effective g-factors and their anisotropy agrees better with the results of the ab initio calculations obtained for the second coordination sphere {[M(fpo)_2_(H_2_O)_2_](ClO_4_)_4_}^2−^, which confirms the existence of the easy-axis anisotropy in 2 (Table S2). Furthermore, this result is supported by the presence of the Orbach relaxation process with an energy barrier close to the Δ_1–2_ (vide infra).

### 2.5. Theoretical Calculations

**Figure 6:**
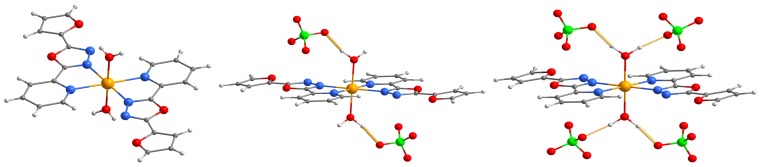
Structures used in calculations of ZFS parameters, molecular fragment [M(fpo)_2_(H_2_O)_2_]^2+^ (left), {[M(fpo)_2_(H_2_O)_2_](ClO_4_)_2_} (middle), and {[M(fpo)_2_(H_2_O)_2_](ClO_4_)_4_}^2−^ (right).

**Figure 7:**
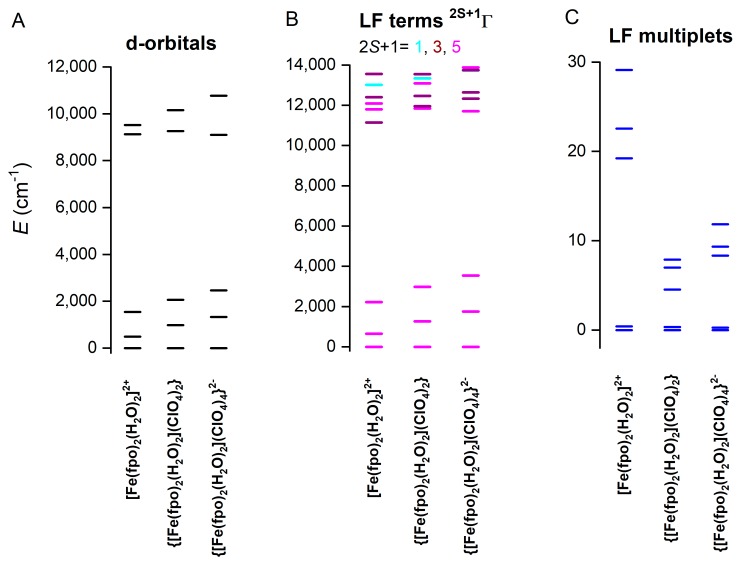
The graphical output of the CASSCF/NEVPT2 calculations with CAS(6,5) for the mononuclear molecular fragments of 1. Plot of the d-orbitals splitting calculated by the ab initio ligand field theory (AILFT) (**A**), low-lying ligand-field terms (**B**), and ligand-field multiplets—Kramers doublets (**C**). The quintet, triplet, and singlet spin states are shown in distinct colours.

**Figure 8:**
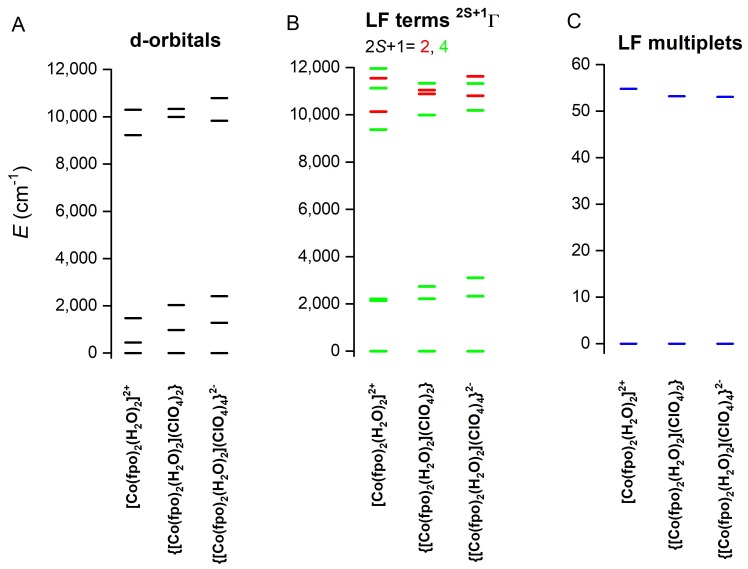
The graphical output of the CASSCF/NEVPT2 calculations with CAS(7,5) for the mononuclear molecular fragments of 2. The plot of the d-orbitals splitting calculated by ab initio ligand field theory (AILFT) (**A**), low-lying ligand-field terms (**B**), and ligand-field multiplets—Kramers doublets (**C**). The doublet and quartet spin states are shown in red and green, respectively.

With the aim to resolve the ambiguity in fitted parameters from DC magnetic data, the multi-reference ab initio calculations based on the state-averaged complete active space self-consistent field method (SA-CASSCF) were performed for both complexes 1 and 2. Herein, the ORCA software package [40,41] was used for these CASSCF calculations with the active space defined as six electrons in five d-orbitals, CAS(6,5) for Fe^II^ complex 1 and seven electrons in five d-orbitals, CAS(7,5), for Co^II^ complex 2. In addition, the dynamic electronic correlation was handled by using the N-electron valence state perturbation theory (NEVPT2) [42,43,44]. These calculations were undertaken on the mononuclear molecular fragments [M(fpo)_2_(H_2_O)_2_]^2+^ extracted from the experimental X-ray structures in which the atomic positions of all hydrogen atoms were optimized using the BP86 functional [45,46,47] together with the atom-pairwise dispersion correction method (D3BJ) [48,49]. The results of CASSCF/NEVPT2 calculations are summarized in Table 2 and Table 3. The calculated ZFS values are far from the experiment, *D* = −6.02 cm^−1^, *E*/*D* = 0.077 vs. for *D* = −1.23 cm^−1^, *E*/*D* = 0.31 for 1, and *D* = 27.2 cm^−1^, *E*/*D* = 0.076 vs. *D* = −21.2 cm^−1^, *E*/*D* = 0.32 for 2. Thus, we employed the double-shell effect by enlarging the active space to 10 d-orbitals, having CAS(6,10) for 1 and CAS(7,10) for 2, but the ZFS parameters changed only slightly. From inspecting the X-ray structures, it is evident that hydrogen bonds are present among aqua ligands and perchlorate anions in the solid-state, which forms the second coordination sphere. The perchlorate anions are present in two different distances. Thus, first, the molecular fragments {[M(fpo)_2_(H_2_O)_2_](ClO_4_)_2_} were considered with shorter O…O distances between the aqua ligand and the perchlorate anion. Again, the analogous calculations were done, and much better agreement with the experimental ZFS parameters was found, *D* = −1.83 cm^−1^, *E*/*D* = 0.223 vs. for *D* = −1.23 cm^−1^, *E*/*D* = 0.31 for 1, and *D* = 23.3 cm^−1^, *E*/*D* = 0.318 vs. *D* = −21.2 cm^−1^, *E*/*D* = 0.32 for 2. Next, the larger second coordination sphere was implemented with four perchlorate anions, {[M(fpo)_2_(H_2_O)_2_](ClO_4_)_4_}^2−^, which resulted only in much smaller changes in ZFS parameters than in the previous case. Figure 6 shows molecular fragments employed for calculations. All this points to the importance of the hydrogen bonds in a solid state in the molecular magnetism. To better visualize the impact of the second coordination sphere on the electronic structure and ZFS, the ab initio ligand field theory (AILFT) was utilized to calculate the energies of the d-orbitals in 1 and 2, as depicted in Figure 7 and Figure 8. Evidently, the change of the molecular fragments {[M(fpo)_2_(H_2_O)_2_]^2+^ → {[M(fpo)_2_(H_2_O)_2_](ClO_4_)_2_} → {[M(fpo)_2_(H_2_O)_2_](ClO_4_)_4_}^2−^ has a very significant impact on d-orbital’s splitting, which is far from ideal O_h_ symmetry. This is reflected in the energy levels of the ligand-field terms and, as expected, in ligand-field multiplets showing the zero-field splitting pattern (Figure 7 and Figure 8). The importance and the impact of the second coordination sphere on the magnetism was also recently studied in other Co(II) complexes [50,51,52].

**Figure 9:**
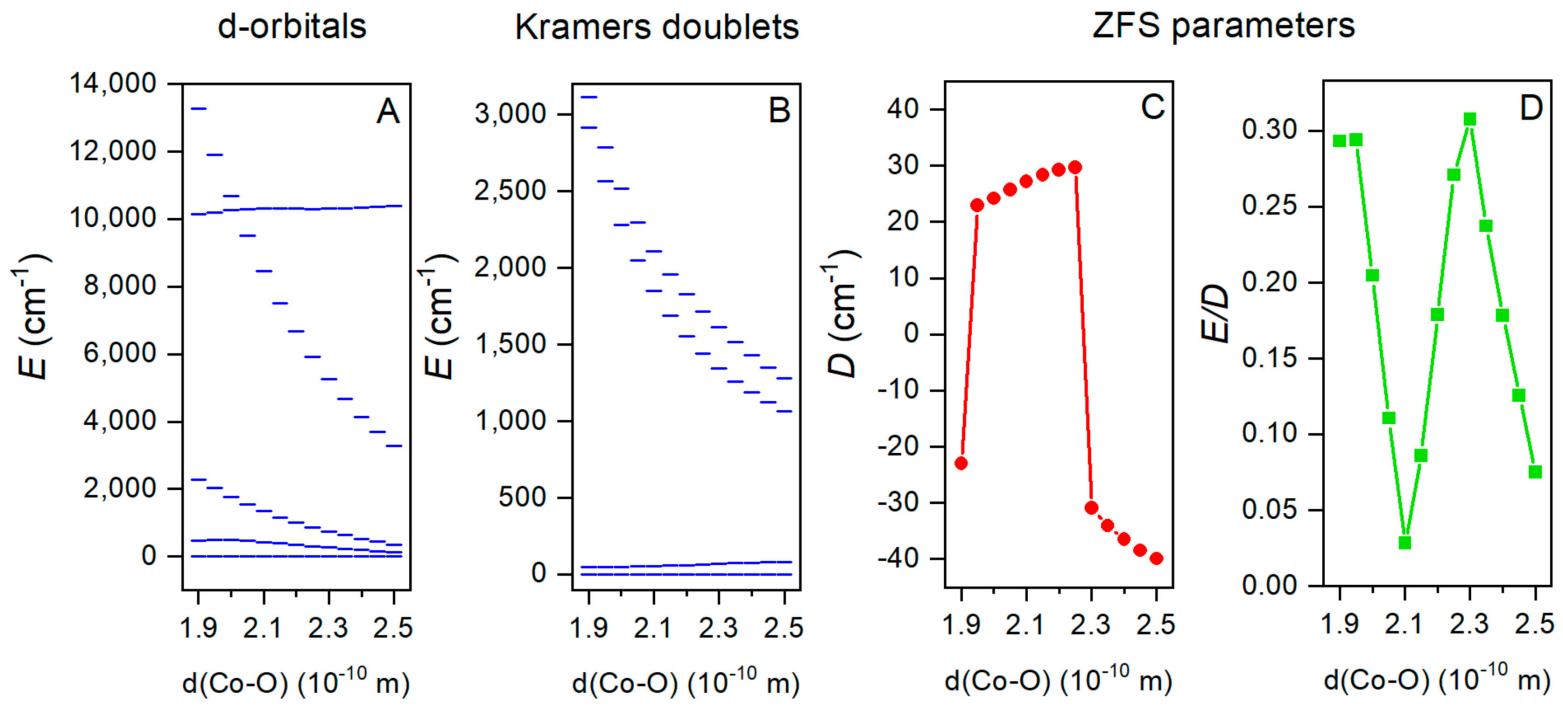
The graphical output of the CASSCF/NEVPT2 calculations with CAS(7,5) for the mononuclear molecular fragments of [Co(fpo)_2_(H_2_O)_2_]^2+^ of **2** for varying d(Co-O) bond distances. The plot of the d-orbitals splitting calculated by ab initio ligand field theory (AILFT) (**A**), four low-lying Kramers doublets (**B**), and the zero-field splitting parameters *D* and *E* (**C** and **D**).

In other words, the perchlorate induced hydrogen bonding alters the axial ligand field of aqua ligands, and, therefore, we decided to model this phenomenon in an alternative way. Thus, the bond distances Co-O in [Co(fpo)_2_(H_2_O)_2_]^2+^ varied from 1.9 to 2.5 Å and, for each geometry, the CASSCF/NEVPT2 calculations with CAS(7,5) were performed. The results of these calculations are shown in Figure 9. Evidently, the stronger axial ligand field led to small |*D*| values. On the contrary, a weak axial ligand field resulted in a negative *D*-parameter with small rhombicity. Generally, the magnetic anisotropy in the modelled system is very sensitive to small changes in the axial ligand field, which is manifested in abrupt changes of the rhombicity.

## 3. Materials and Methods

Chemicals for syntheses were purchased from Sigma-Aldrich (St. Louis, MO, USA), Across Organics (Geel, Belgium), or Alfa Aesar (Kandel, Germany) and used without any further purification. Reactions were monitored on aluminium TLC sheets pre-coated with silica gel 60 (SIL G/UV_254_, 0.2 mm, Macherey-Nagel, Düren, Germany). Ligand fpo was purified by column chromatography on Merck silica gel 60 (0.015–0.040 nm, Darmstadt, Germany) and the reaction scheme of organic syntheses drawn by a BIOVIA draw [53]. Elemental analysis of chemical composition was acquired by the Flash 2000 (Thermo Scientific, Waltham, MA, USA) analyser. IR measurements were performed on spectrometer Jasco FT/IR-4700, data interpreted in Spectragryph, and assigned with the help of the table of known characteristic vibration frequencies [54,55]. NMR (Varian, Palo Alto, CA, USA) experiments ^1^H and ^13^C were conducted on 400 MHz Varian spectrometer using CDCl_3_ or *d6*-DMSO solvent and processed in the iNMR program [56]. Magnetic measurements on PPMS Dynacool (Quantum Design, Quantum Design, San Diego, CA, USA) and SQUID magnetometer XL-7 (Quantum Design). X-ray experiments were carried out on a four-circle κ-axis Xcalibur2 diffractometer equipped with a CCD detector Sapphire2 (Rigaku Oxford Diffraction, Yarnton, UK). The CrysAlis software package (version 1.171.39.9g, Rigaku Oxford Diffraction, Yarnton, United Kingdom) was used for data collection and reduction [57]. The structures for both 1 and 2 were solved by the SHELXT program incorporated in the wingx program package [58,59]. Refinement based on intensities was performed using the SHELXL program [60]. All non-hydrogen atoms in both 1 and 2 were refined anisotropically. Perchlorate anions in both compounds were found to be disordered in two positions and the ratio between both parts was 0.83:0.17 for 1 and 0.82:0.18 for 2. Hydrogen atoms in the ligand were placed in the calculated positions with isotropic thermal parameters tied with the parent atoms (U(H) = 1.2U(C)). The positions of the water hydrogen atoms for the aqua ligands were found in different Fourier maps and their isotropic thermal parameters were tied with the parent atoms (U(H) = 1.2U(O)). The structural figures were drawn using the Diamond software [61]. Crystal data and the final parameters of the structural refinements for both 1 and 2 are summarized in Table 1. The EPR spectra were studied using Bruker ELEXSYS II E500 X–band spectrometer (Bruker BioSpin GmbH, Rheinstetten, Germany) with an operating frequency of 9.4 GHz equipped with ESR910 helium flow–type cryostat (Oxford Instruments plc, Abingdon, UK). The powdered sample was mixed with Apiezon N grease (M&I Materials Ltd, Manchester, UK) and attached to the Suprasil sample holder (Wilmad-LabGlass, Vineland, NJ, USA).

### 3.1. Preparation of Organic Ligands

#### 3.1.1. Methyl Picolinate (I)

Picolinic acid (10 g, 41.2 mmol) was put to 100 mL of methanol followed by careful addition of 12 mL concentrated sulphuric acid. Heating the mixture resulted in dissolving picolinic acid. After one day of reflux, the solution was concentrated, poured into 400 mL of water and ice mixture, neutralized with potassium bicarbonate, and extracted five times with 50 mL of chloroform and dried with MgSO_4_. Evaporation of chloroform and drying under vacuum for a few hours yielded 93% of I as a slightly yellow oil, which was used in a subsequent reaction without further purification. FT-IR (mid ATR, *v*/cm^−1^): 3058 *v*(C-H_arom_), 2952 *v*(CH_3_), 2844 *v*(CH_3_), 1742 *v*(CO), 1719 *v*(CO), 1582 *v*(C=C, C=N), 1572 *v*(C=C, C=N), 1469 *v*(C=C, C=N), 1442 (CH_3_), 1431 *v*(C=C, C=N), 1281 *v*(COC), 1244 *v*(OCH_3_), 1125 *v*(COC), 995 *v*(ring, pyridine), 745 *v*(C-H_arom_), 705 *v*(C-H_arom_), 619 *v*(ring, pyridine), 405 *v*(ring, pyridine). ^1^H NMR (400 MHz, CDCl_3_) *δ* 8.70 (ddd, *J* = 4.7, 1.7, 0.9 Hz, 1H), 8.09 (dt, *J* = 7.8, 1.0 Hz, 1H), 7.80 (td, *J* = 7.8, 1.7 Hz, 1H), 7.43 (ddd, *J* = 7.6, 4.8, 1.2 Hz, 1H), 3.96 (s, 3H). ^13^C NMR (101 MHz, CDCl_3_) *δ* 165.73, 149.85, 147.92, 137.12, 127.03, 125.19, 52.97.

#### 3.1.2. Picolinic Acid Hydrazide (II)

Compound I (4.53 g, 33 mmol) was poured into 75 mL of methanolic solution containing 50%–60% hydrazine hydrate (8.4 g) and the mixture was refluxed for 14 h. Subsequent volume reduction of solvent and cooling of flask with the product in ice results in the formation of colourless crystalline solid of II. Further purification was accomplished by dissolving II in hot toluene and quick cooling by ice. This procedure induces formation of elongated transparent colourless crystals and yield 78%. EA (calc.%) for C_6_H_7_N_3_O (FW = 137.14): C, 52.55, H, 5.14, N, 30.64, Found: C, 52.34, H, 5.36, N, 30.49. FT-IR (mid ATR, *v*/cm^−1^): 3288 *v*(N-H), 3210 *v*(NH), 3017 *v*(C-H_arom_), 1671 *v*(C=O), 1622 *v*(NH_2_), 1593 *v*(C=C, C=N), 1568 *v*(C=C, C=N), 1518 *v*(C=C, C=N, NH_2_), 1433 *v*(C=C, C=N), 1123 *v*(C-N), 999 *v*(ring, pyridine), 819 *v*(C-H_arom_), 750 *v*(C-H_arom_), 620 *v*(ring, pyridine), 410 *v*(ring, pyridine). ^1^H NMR (400 MHz, DMSO-*d*6) *δ* 9.9 (s, 1H), 8.6 (m, 1H), 8.01–7.93 (m, 2H), 7.58-7.53 (m, 1H), 4.61 (s, 2H). ^13^C NMR (101 MHz, DMSO-*d*6) *δ* 162.68, 149.86, 148.52, 137.67, 126.27, 121.76.

#### 3.1.3. Picolinic Acid 2-(2-furanylmethylene)hydrazide (III)

Furfural (1.5 g, 16 mmol) in 10 mL of methanol was added to II (2 g, 14.6 mmol) in 20 mL of methanol. After 2 h of boiling the solution, cooling in ice caused colourless crystals to form quickly from a yellow to an orange solution. Reducing the volume of methanol to a minimum amount (about one-fifth) yielded an additional amount of product. Repeated washing of crystals with a small amount of cold methanol yields a pure product (94%). EA (calc.%) for C_11_H_9_N_3_O_2_ (FW = 215.21): C, 61.39, H, 4.22, N, 19.53, Found: C, 61.31, H, 4.12, N, 19.54. FT-IR (mid ATR, *v*/cm^−1^): 3287 *v*(NH), 3225 *v*(NH), 3132 *v*(C-H_arom_), 3112 *v*(C-H_arom_), 3086 *v*(C-H_arom_), 3060 *v*(C-H_arom_), 3003 *v*(C-H_arom_), 1675 *v*(C=O), 1627 *v*(C=N, imine), 1590 *v*(C=C, C=N), 1572 *v*(C=C, C=N), 1520 *v*(C=C, C=N), 1434 *v*(C=C, C=N), 996 *v*(ring, pyridine), 613 *v*(ring, pyridine), 594 (ring, furan), and 406 *v*(ring, pyridine). ^1^H NMR (400 MHz, DMSO-*d*6) δ 12.23 (s, 1H), 8.70 (ddd, *J* = 4.7, 1.5, 1.0 Hz, 1H), 8.56 (s, 1H), 8.12 (dt, *J* = 7.8, 1.0 Hz, 1H), 8.05 (td, *J* = 7.8, 1.7 Hz, 1H), 7.86 (d, *J* = 1.7 Hz, 1H), 7.66 (ddd, *J* = 7.5, 4.7, 1.3 Hz, 1H), 6.92 (d, *J* = 3.4 Hz, 1H), 6.64 (dd, *J* = 3.4, 1.8 Hz, 1H). ^13^C NMR (101 MHz, DMSO-*d*6) δ 160.40, 149.57, 149.51, 148.48, 145.30, 138.78, 138.06, 127.04, 122.73, 113.59, 112.30.

#### 3.1.4. 2-(furan-2-yl)-5-(pyridin-2-yl)-1,3,4-oxadiazole (fpo)

A clear solution of III (500 mg, 2.32 mmol) in 10 mL of dimethylsulfoxide was stirred at room temperature and potassium carbonate (1284 mg, 9.29 mmol) with iodine (1062 mg, 4.18 mmol) were slowly added. Dark solution formed and temperature was adjusted to 120 °C for 18 h (TLC confirmed formation of fpo and consumed starting material III, TLC mobile phase cyclohexane:ethylacetate = 2:1). Afterward, dimethylsulfoxide was evaporated. The solid was dissolved in water containing sodium metabisulfite and extracted three times with 20 mL of ethylacetate. Combined parts of ethylacetate were evaporated and re-dissolved in a minimum amount of cyclohexane:ethylacetate (2:1) and subjected to column chromatography on silica gel with gradient elution (cyclohexane:ethylacetate ≥ 5:1, 3:1 and 2:1). The final product fpo precipitated as white solid in 62% yield after evaporating solvent from joined parts. Colourless crystals can be obtained by slowly removing cyclohexane/ethylacetate. EA (calc.%) for C_11_H_7_N_3_O_2_ (FW = 213.19): C, 61.97, H, 3.31, N, 19.71. Found: C, 61.96, H, 3.01, N, 19.46. FT-IR (mid ATR, *v*/cm^−1^): 3128 *v*(C-H_arom_), 3107 *v*(C-H_arom_), 3092 *v*(C-H_arom_), 3057 *v*(C-H_arom_), 3025 *v*(C-H_arom_), 3000 *v*(C-H_arom_), 1615 (C=C, C=N), 1587 (C=C, C=N), 1574 (C=C, C=N), 1525 (C=C, C=N), 1421 *v*(C=C, C=N), 995 *v*(ring, pyridine), 619 *v*(ring, pyridine), 593 (ring, furan), 400 *v*(ring, pyridine). ^1^H NMR (400 MHz, DMSO-*d*6) *δ* 8.79 (ddd, *J* = 4.8, 1.7, 1.0 Hz, 1H), 8.23 (dt, *J* = 7.8, 1.0 Hz, 1H), 8.11 (dd, *J* = 1.8, 0.7 Hz, 1H), 8.06 (td, *J* = 7.8, 1.7 Hz, 1H), 7.64 (ddd, *J* = 7.6, 4.8, 1.2 Hz, 1H), 7.47 (dd, *J* = 3.6, 0.7 Hz, 1H), 6.83 (dd, *J* = 3.6, 1.8 Hz, 1H). ^13^C NMR (101 MHz, DMSO-*d*6) *δ* 162.76, 157.56, 150.30, 147.30, 142.50, 138.48, 137.88, 126.47, 123.13, 115.17, 112.80.

### 3.2. Preparation of the Complexes

Preparation of coordination compounds was performed as follows: Fe(II) (with a small amount of ascorbic acid) or Co(II) perchlorate hexahydrate (0.234 mmol) in 10 mL of methanol was added dropwise to fpo (100 mg, 0.469 mmol) in 10 mL of methanol. After an hour of reflux, the solution was filtered and left to evaporate slowly. Square shaped crystals formed within one week.

**Caution!** Work with perchlorate salts may be dangerous because of the potentially explosive character!

[Fe(fpo)_2_(H_2_O)_2_](ClO_4_)_2_ (**1**). Yellow crystals, 52 mg (31%). EA (calc.%) for C_22_H_18_Cl_2_FeN_6_O_14_ (FW = 717.17): C, 36.84, H, 2.53, N, 11.72, Found: C, 37.06, H, 2.18, N, 11.36. FT-IR (mid ATR, *v*/cm^−1^): 3363 *v*(H_2_O), 3124 *v*(C-H_arom_), 3070 *v*(C-H_arom_), 1634 *v*(H_2_O), 1575 (C=C, C=N), 1519 (C=C, C=N), 1422 *v*(C=C, C=N), 1094 *v*(ClO_4_), 1048 *v*(ClO_4_), 623 *v*(ClO_4_), 593 (ring, furan), 414 *v*(ring, pyridine).

[Co(fpo)_2_(H_2_O)_2_](ClO_4_)_2_ (**2**). Orange crystals, 40.7 mg (24%). EA (calc.%) for C_22_H_18_Cl_2_CoN_6_O_14_ (FW = 720.25): C, 36.69, H, 2.52, N, 11.67. Found: C, 36.80, H, 2.45, N, 11.43. FT-IR (mid ATR, *v*/cm^−1^): 3384 *v*(H_2_O), 3124 *v*(C-H_arom_), 3072 *v*(C-H_arom_), 1633 *v*(H_2_O), 1574 (C=C, C=N), 1517 (C=C, C=N), 1423 *v*(C=C, C=N), 1094 *v*(ClO_4_), 1049 *v*(ClO_4_), 623 *v*(ClO_4_), 592 (ring, furan), 419 *v*(ring, pyridine).

## 4. Conclusions

Our work focused on preparation and magneto-chemical analysis of new 1,3,4-oxadiazole based coordination compounds. Thus, isolated coordination compounds [M(fpo)_2_(H_2_O)_2_](ClO_4_)_2_ (M = Fe(II) for (1); Co(II) for (2)) may serve as potential building blocks for preparation of polynuclear compounds. The data showed large rhombicity in both complexes and, in the case of 2, field-induced slow relaxation of the magnetization with an energy barrier *U*_eff_ = 65.3 K was detected. The axial type of the magnetic anisotropy was also confirmed in 2 by employing X-band EPR. Detailed theoretical investigation based on the CASSCF/NEVPT2 calculations revealed the importance of the second coordination sphere formed by the hydrogen bonding between perchlorate anions and aqua ligands on the magnetic anisotropy parameters D and E. The hydrogen bond tunes the axial ligand field and, thus, this kind of non-covalent contacts can serve as a trigger between the easy-plane and the easy-axis type of the magnetic anisotropy.

## Figures and Tables

**Table 1:** Crystallographic data and structure refinement parameters of the compounds **1** and **2**.

Compound	1	2
Chemical formula	C_22_H_18_Cl_2_FeN_6_O_14_	C_22_H_18_Cl_2_CoN_6_O_14_
Formula weight	717.17	720.25
Temperature [K]	293(2)	293(2)
*λ* (Mo, Kα) [Å]	0.71073	0.71073
Crystal system	monoclinic	monoclinic
Space group	*P*2_1_/*c*	*P*2_1_/*c*
*a* [Å]	8.8543(18)	8.8827(9)
*b* [Å]	13.627(3)	13.6502(12)
*c* [Å]	11.840(2)	11.7754(10)
*α* [°]	90	90
*β* [°]	91.26(3)	91.469(7)
*γ* [°]	90	90
Volume [Å^3^]	1428.2(5)	1427.3(2)
Z	2	2
*D* [g·cm^−3^]	1.668	1.662
*μ* [mm^−1^]	0.80	0.86
*F* (000)	728	726
Reflections collected	5761	5605
Independent reflections	3198	3165
*R*_int_	0.017	0.065
*R*[*F*^2^ > 2*σ*(*F*^2^)]	0.049	0.072
*wR*(*F*^2^)	0.133	0.215
Σ	1.02	1.02

**Table 2:** Comparison of the spin Hamiltonian parameters for 1 derived from CASSCF/NEVPT2 calculations done in ORCA and the experimental magnetic data ^a^.

	*D*	*E/D*	*g*_1_	*g*_2_	*g*_3_	δ ^b^	Δ_1–5_^c^	Δ_1–6_^c^
	**[Fe(fpo)_2_(H_2_O)_2_]^2+^**							
CAS(6,5)	−7.05	0.077	2.045	2.076	2.234	654	29.1	679
CAS(6,10)	−6.02	0.082	2.045	2.072	2.225	623	24.9	645
	**{[Fe(fpo)_2_(H_2_O)_2_](ClO_4_)_2_}**							
CAS(6,5)	−1.83	0.223	2.055	2.084	2.110	1267	7.90	1280
CAS(6,10)	−2.04	0.158	2.054	2.079	2.104	1238	6.26	1196
	**{[Fe(fpo)_2_(H_2_O)_2_](ClO_4_)_4_}^2−^**							
CAS(6,5)	−2.91	0.060	2.058	2.073	2.081	1761	11.9	1773
CAS(6,10)	−3.06	0.061	2.057	2.069	2.076	1727	12.5	1739
	***Experimentally Determined Parameters***							
	+1.24	0.32	2.094	2.094	2.094			
	−1.23	0.31	2.094	2.094	2.094			

^a^ The values of the parameters are in cm^−1^. ^b^ δ is the energy of the first excited ligand field term. ^c^ The Δ_1–5_ and Δ_1–6_ are energies of the fifth and sixth spin levels, respectively.

**Table 3:** Comparison of the spin Hamiltonian parameters for 2 derived from CASSCF/NEVPT2 calculations done in ORCA and the experimental magnetic data ^a^.

	*D*	*E/D*	g_1_	*g*_2_	*g*_3_	δ ^b^	Δ_1–4_^c^	Δ_1–5_^c^
	**[Co(fpo)_2_(H_2_O)_2_]^2+^**							
CAS(7,5)	27.2	0.076	2.090	2.352	2.374	2138	54.8	1964
CAS(7,10)	27.1	0.084	2.091	2.356	2.383	1967	54.7	1825
	**{[Co(fpo)_2_(H_2_O)_2_](ClO_4_)_2_}**							
CAS(7,5)	23.3	0.318	2.092	2.283	2.385	2223	53.2	2187
CAS(7,10)	23.3	0.307	2.093	2.291	2.392	2068	52.8	2031
	**{[Co(fpo)_2_(H_2_O)_2_](ClO_4_)_4_}^2−^**							
CAS(7,5)	−24.9	0.212	2.096	2.242	2.390	2331	53.1	2331
CAS(7,10)	−24.4	0.222	2.098	2.249	2.396	2160	52.3	2160
	*Experimentally determined parameters*							
	+18.9	0.33	2.31	2.31	2.02			
	−21.2	0.32	2.13	2.13	2.37			

^a^ The values of the parameters are in cm^−1^. ^b^ δ is the energy of the first excited ligand field term. ^c^ The Δ_1–4_ and Δ_1–5_ are energies of the fourth and fifth spin levels, respectively.

## Supplementary Materials

The following are available online at https://www.mdpi.com/1420-3049/25/2/277/s1. Figure S1: In-phase χ_real_ and out-of-phase χ_imag_ molar susceptibilities for 2 at zero static magnetic field and in non-zero static field. Table S1: Parameters of one-component Debye model for 2 derived according Eq.4 in main text. Table S2: The g-values for the ground state Kramers doublet calculated for the effective spin *S*_eff_ = 1/2 for 2 derived from CASSCF/NEVPT2 calculations. CCDC 1972873-1972874 contain the supplementary crystallographic data for this paper.

## References

[1] Bartolomé J., Luis F., Fernández J.F. (2016). Molecular Magnets: Physics and Applications.

[2] Ardavan A., Rival O., Morton J.J.L., Blundell S.J., Tyryshkin A.M., Timco G.A., Winpenny R.E.P. (2007). Will spin-relaxation times in molecular magnets permit quantum information processing?. Phys. Rev. Lett..

[3] Kahn O. (1998). Spin-transition polymers: From molecular materials toward memory devices. Science.

[4] Bartual-Murgui C., Akou A., Thibault C., Molnár G., Vieu C., Salmon L., Bousseksou A. (2015). Spin-crossover metal–organic frameworks: Promising materials for designing gas sensors. J. Mater. Chem. C.

[5] Linares J., Codjovi E., Garcia Y. (2012). Pressure and temperature spin crossover sensors with optical detection. Sensors.

[6] Tripathi S., Dey A., Shanmugam M., Narayanan R.S., Chandrasekhar V., Chandrasekhar V., Pointillart F. (2018). Cobalt(II) Complexes as Single-Ion Magnets. Organometallic Magnets. Topics in Organometallic Chemistry.

[7] Halcrow M.A. (2013). Spin-Crossover Materials: Properties and Applications.

[8] Yao X.-N., Du J.-Z., Zhang Y.-Q., Leng X.-B., Yang M.-W., Jiang S.-D., Wang Z.-X., Ouyang Z.-W., Deng L., Wang B.-W. (2016). Two-coordinate Co(II) imido complexes as outstanding single-molecule magnets. J. Am. Chem. Soc..

[9] Böhme M., Ziegenbalg S., Aliabadi A., Schnegg A., Görls H., Plass W. (2018). Magnetic relaxation in cobalt(ii)-based single-ion magnets influenced by distortion of the pseudotetrahedral [N2O2] coordination environment. Dalton Trans..

[10] Chandrasekhar V., Dey A., Mota A.J., Colacio E. (2013). Slow magnetic relaxation in Co(III)–Co(II) mixed-valence dinuclear complexes with a CoIIO5X (X = Cl, Br, NO_3_) distorted-octahedral coordination sphere. Inorg. Chem..

[11] Kobayashi F., Ohtani R., Nakamura M., Lindoy L.F., Hayami S. (2019). Slow magnetic relaxation triggered by a structural phase transition in long-chain-alkylated cobalt(II) single-ion magnets. Inorg. Chem..

[12] Halcrow M.A. (2011). Structure:function relationships in molecular spin-crossover complexes. Chem. Soc. Rev..

[13] Gütlich P., Gaspar A.B., Garcia Y. (2013). Spin state switching in iron coordination compounds. Beilstein J. Org. Chem..

[14] Bar A.K., Pichon C., Sutter J.-P. (2016). Magnetic anisotropy in two—to eight-coordinated transition–metal complexes: Recent developments in molecular magnetism. Coord. Chem. Rev..

[15] Herchel R., Váhovská L., Potočňák I., Trávníček Z. (2014). Slow magnetic relaxation in octahedral cobalt(II) field-induced single-ion magnet with positive axial and large rhombic anisotropy. Inorg. Chem..

[16] Váhovská L., Vitushkina S., Potočňák I., Trávníček Z., Herchel R. (2018). Effect of linear and non-linear pseudohalides on the structural and magnetic properties of Co(II) hexacoordinate single-molecule magnets. Dalton Trans..

[17] Váhovská L., Bukrynov O., Potočňák I., Čižmár E., Kliuikov A., Vitushkina S., Dušek M., Herchel R. (2018). New cobalt(II) field-induced single-molecule magnet and the first example of a cobalt(III) complex with tridentate binding of a deprotonated 4-amino-3,5-bis(pyridin-2-yl)-1,2,4-triazole ligand. Eur. J. Inorg. Chem..

[18] Du M., Wang Q., Li C.-P., Zhao X.-J., Ribas J. (2010). Coordination assemblies of CoII/CuII/ZnII/CdIIwith 2,5-bipyridyl-1,3,4-oxadiazole and dicyanamide anion: Structural diversification and properties. Cryst. Growth Des..

[19] Li C.-P., Zhao X.-H., Chen X.-D., Yu Q., Du M. (2010). Metal-involved solvothermal interconversions of pyrazinyl substituted azole derivatives: Controllability and mechanism. Crys. Growth Des..

[20] Gudasi K., Patil M., Vadavi R., Shenoy R., Patil S. (2007). Transition metal complexes with a new tridentate ligand, 5-[6-(5-mercapto- 1,3,4-oxadiazol-2-yl)pyridin-2-yl]-1,3,4-oxadiazole-2-thiol. J. Serb. Chem. Soc..

[21] Mathew G., Suseelan M.S., Krishnan R. (2006). Synthesis and characterization of cobalt (II) complexes of chromen-2-one-3-carboxy hydrazide and 2-(chromen-2′-onyl)-5-(aryl) 1,3,4-oxadiazole derivatives. Indian J. Chem. A.

[22] Wang Y.-T., Tang G.-M., Ma W.-Y., Wan W.-Z. (2007). Synthesis and characterization of two new coordination supramolecular structures from a versatile unsymmetric 5-(4-pyridyl)-1,3,4-oxadiazole-2-thione (Hpot) ligand. Polyhedron.

[23] Incarvito C., Rheingold A.L., Qin C.J., Gavrilova A.L., Bosnich B. (2001). Bimetallic reactivity. On the use of oxadiazoles as binucleating ligands. Inorg. Chem..

[24] Incarvito C., Rheingold A.L., Gavrilova A.L., Qin C.J., Bosnich B. (2001). Bimetallic reactivity. One-site addition Two-metal oxidation reactions using a Di-Co(II) complex of a binucleating ligand with 5-and 6-coordinate sites. Inorg. Chem..

[25] Subha C., Selvaraj A. (2014). Synthesis and characterization of Co (II) And Ni (II) complexes of 2, 5-substituted 1, 3, 4-oxadiazole derivatives. Res. J. Chem. Sci..

[26] Goswami S., Jena H.S., Konar S. (2014). Study of Heterogeneous Catalysis by Iron-squarate based 3D metal organic framework for the transformation of tetrazines to oxadiazole derivatives. Inorg. Chem..

[27] Klingele J., Kaase D., Schmucker M., Lan Y., Chastanet G., Létard J.-F. (2013). Thermal spin crossover and LIESST effect observed in complexes [Fe(LCh)2(NCX)2] [LCh = 2,5-Di(2-pyridyl)-1,3,4-Chalcadiazole; Ch = O, S, Se; X = S, Se, BH3]. Inorg. Chem..

[28] Köhler C., Rentschler E. (2015). The first 1,3,4-oxadiazole based dinuclear iron(II) complexes showing spin crossover behavior with hysteresis. Eur. J. Inorg. Chem..

[29] Phung Q.M., Domingo A., Pierloot K. (2017). Dinuclear iron(II) spin-crossover compounds: A theoretical study. Chem. Eur. J..

[30] Mani G.S., Donthiboina K., Shankaraiah N., Kamal A. (2019). Iodine-promoted one-pot synthesis of 1,3,4-oxadiazole scaffolds via sp3 C–H functionalization of azaarenes. New J. Chem..

[31] Lu G.-P., Lin Y.-M. (2014). A base-induced ring-opening process of 2-substituted-1,3,4-oxadiazoles for the generation of nitriles at room temperature. J. Chem. Res..

[32] Yu W., Huang G., Zhang Y., Liu H., Dong L., Yu X., Li Y., Chang J. (2013). I2-mediated oxidative C–O bond formation for the synthesis of 1,3,4-oxadiazoles from aldehydes and hydrazides. J. Org. Chem..

[33] Dijken D.J.V., Kovaříček P., Ihrig S.P., Hecht S. (2015). Acylhydrazones as widely tunable photoswitches. J. Am. Chem. Soc..

[34] Boča R. (1999). Theoretical Foundations of Molecular Magnetism.

[35] Zhang Y.-Z., Gómez-Coca S., Brown A.J., Saber M.R., Zhang X., Dunbar K.R. (2016). Trigonal antiprismatic Co(ii) single molecule magnets with large uniaxial anisotropies: Importance of Raman and tunneling mechanisms. Chem. Sci..

[36] Stoll S., Schweiger A. (2006). EasySpin, a comprehensive software package for spectral simulation and analysis in EPR. J. Magn. Reson..

[37] Boča R., Mingos D.M.P. (2005). Magnetic Parameters and Magnetic Functions in Mononuclear Complexes Beyond the Spin-Hamiltonian Formalism. Structure and Bonding.

[38] Palii A.V., Korchagin D.V., Yureva E.A., Akimov A.V., Misochko E.Y., Shilov G.V., Talantsev A.D., Morgunov R.B., Aldoshin S.M., Tsukerblat B.S. (2016). Single-ion magnet Et4N[CoII(hfac)3] with nonuniaxial Anisotropy: Synthesis, experimental characterization, and theoretical modeling. Inorg. Chem..

[39] Titiš J., Boča R. (2011). Magnetostructuraldcorrelations in hexacoordinated cobalt(II) complexes. Inorg. Chem..

[40] Neese F. (2012). The ORCA program system. Wiley Interdiscip. Rev. Comput. Mol. Sci..

[41] Neese F. (2018). Software update: The ORCA program system, version 4.0. Wiley Interdiscip. Rev. Comput. Mol. Sci..

[42] Angeli C., Cimiraglia R., Malrieu J.P. (2001). N-electron valence state perturbation theory: A fast implementation of the strongly contracted variant. Chem. Phys. Lett..

[43] Angeli C., Cimiraglia R., Evangelisti S., Leininger T., Malrieu J.P. (2001). Introduction of n-electron valence states for multireference perturbation theory. J. Chem. Phys..

[44] Angeli C., Cimiraglia R., Malrieu J.P. (2002). n-electron valence state perturbation theory: A spinless formulation and an efficient implementation of the strongly contracted and of the partially contracted variants. J. Chem. Phys..

[45] Becke A.D. (1988). Density-functional exchange-energy approximation with correct asymptotic behavior. Phys. Rev. A.

[46] Perdew J.P. (1986). Density-functional approximation for the correlation energy of the inhomogeneous electron gas. Phys. Rev. B Condens. Matter Mater. Phys..

[47] Perdew J.P. (1986). Erratum: Density-functional approximation for the correlation energy of the inhomogeneous electron gas. Phys. Rev. B Condens. Matter Mater. Phys..

[48] Grimme S., Ehrlich S., Goerigk L. (2011). Effect of the damping function in dispersion corrected density functional theory. J. Comput. Chem..

[49] Grimme S., Antony J., Ehrlich S., Krieg H. (2010). A consistent and accurate ab initio parametrization of density functional dispersion correction (DFT-D) for the 94 elements H-Pu. J. Chem. Phys..

[50] Suturina E.A., Maganas D., Bill E., Atanasov M., Neese F. (2015). Magneto-structural correlations in a series of pseudotetrahedral [CoII(XR)4]2–single molecule magnets: An ab initio ligand field study. Inorg. Chem..

[51] Petit S., Pilet G., Luneau D., Chibotaru L.F., Ungur L. (2007). A dinuclear cobalt(ii) complex of calix [8] arenes exibiting strong magnetic anisotropy. Dalton Trans..

[52] Mondal A., Kharwar A.K., Konar S. (2019). Sizeable effect of lattice solvent on field induced slow magnetic relaxation in seven coordinated CoII complexes. Inorg. Chem..

[53] Dassault Systèmes BIOVIA (2019). BIOVIA Draw, Version 19.1.

[54] Menges F. (2019). Spectragryph—Optical Spectroscopy Software, Version 1.2.12. http://www.effemm2.de/spectragryph/.

[55] Socrates G. (2015). Infrared and Raman Characteristic Group Frequencies: Tables and Charts.

[56] iNMR. http://www.inmr.net.

[57] (2015). CrysAlisPro, Version 1.171.39.9g Rigaku Oxford Diffraction. https://www.rigaku.com/en/products/smc/crysalis..

[58] Sheldrick G.M. (2015). SHELXT—Integrated space-group and crystal-structure determination. Acta Crystallogr. Sect. A.

[59] Farrugia L.J. (1999). WinGX suite for small-molecule single-crystal crystallography. J. Appl. Cryst..

[60] Sheldrick G.M. (2015). Crystal structure refinement with SHELXL. Acta Crystallogr. Sect. C.

[61] Brandenburg K., Putz H. (1999). DIAMOND.

