# Supplementary material for: The Structural and Magnetic Properties of FeII and CoII Complexes with 2-(furan-2-yl)-5-pyridin-2-yl-1,3,4-oxadiazole
Source: Molecules. 2020 Jan 9;25(2):277. doi: 10.3390/molecules25020277 (PMC7024161; doi:10.3390/molecules25020277)
Supplement: Supplementary file 1 [file molecules-25-00277-s001.pdf]

Supplementary Materials

The structural and magnetic properties of Fe^{II} and Co^{II} complexes with 2-(furan-2-yl)-5-pyridin-2-yl-1,3,4-oxadiazole

Pavel Zoufalý [1], Erik Čižmár [2], Juraj Kuchár [1,3] and Radovan Herchel [1,*]

[1] Department of Inorganic Chemistry, Faculty of Science, Palacký University, 17. listopadu 12, CZ-771 46 Olomouc, Czech Republic; pavel.zoufaly@upol.cz; juraj.kuchar@upol.cz

[2] P.J. Šafárik University in Košice, Faculty of Science, Institute of Physics, Park Angelinum 9, SK-041 54 Košice, Slovakia ; erik.cizmar@upjs.sk

[3] P.J. Šafárik University in Košice, Faculty of Science, Institute of Chemistry, Department of Inorganic Chemistry, Moyzesova 11, SK-041 54 Košice, Slovakia; juraj.kuchar@upjs.sk

* Correspondence: radovan.herchel@upol.cz; Tel.: +420 58563 4435

Table S1. Parameters of one-component Debye model for 2 derived according Eq.4 in main text.

T/K	χ_S/(10^{-6} m^3mol^{-1})	χ_T/(10^{-6} m^3mol^{-1})	α	τ/s
1.9	0.5414	11.0301	0.21247	5.41E-03
2.1	0.4562	9.8960	0.21788	4.26E-03
2.3	0.4794	9.1067	0.20218	3.59E-03
2.5	0.4587	8.4973	0.20646	3.09E-03
2.7	0.4763	7.8337	0.20290	2.60E-03
2.9	0.4874	7.2190	0.18749	2.18E-03
3.1	0.5046	6.7876	0.17255	1.90E-03
3.3	0.5507	6.3644	0.14523	1.61E-03
3.5	0.4536	6.0045	0.15623	1.27E-03
3.7	0.4878	5.6856	0.12545	1.02E-03
3.9	0.5564	5.4039	0.08493	7.89E-04
4.1	0.4721	5.1482	0.07469	5.26E-04
4.3	0.4780	4.9185	0.05209	3.33E-04
4.5	0.4524	4.7052	0.03086	2.06E-04
4.7	0.4647	4.5190	0.01977	1.27E-04
4.9	0.3985	4.3466	0.01064	7.43E-05
5.1	0.4092	4.1932	0.00967	4.52E-05
5.3	0.5860	4.0464	0.00928	2.82E-05

Table S2. The g-values for the ground state Kramers doublet calculated for the effective spin S_{eff} = 1/2 for **2** derived from CASSCF/NEVPT2 calculations

	g_1	g_2	g_3
	$[Co(fpo)_2(H_2O)_2]^{2+}$		
CAS(7,5)	2.076654	4.160317	5.232077
CAS(7,10)	2.070967	4.103990	5.307797
	$\{[Co(fpo)_2(H_2O)_2](ClO_4)_2\}$		
CAS(7,5)	1.589525	2.364502	6.468165
CAS(7,10)	1.620814	2.437684	6.450145
	$\{[Co(fpo)_2(H_2O)_2](ClO_4)_4\}^{2-}$		
CAS(7,5)	1.141082	1.469279	6.876476
CAS(7,10)	1.182593	1.537428	6.869937

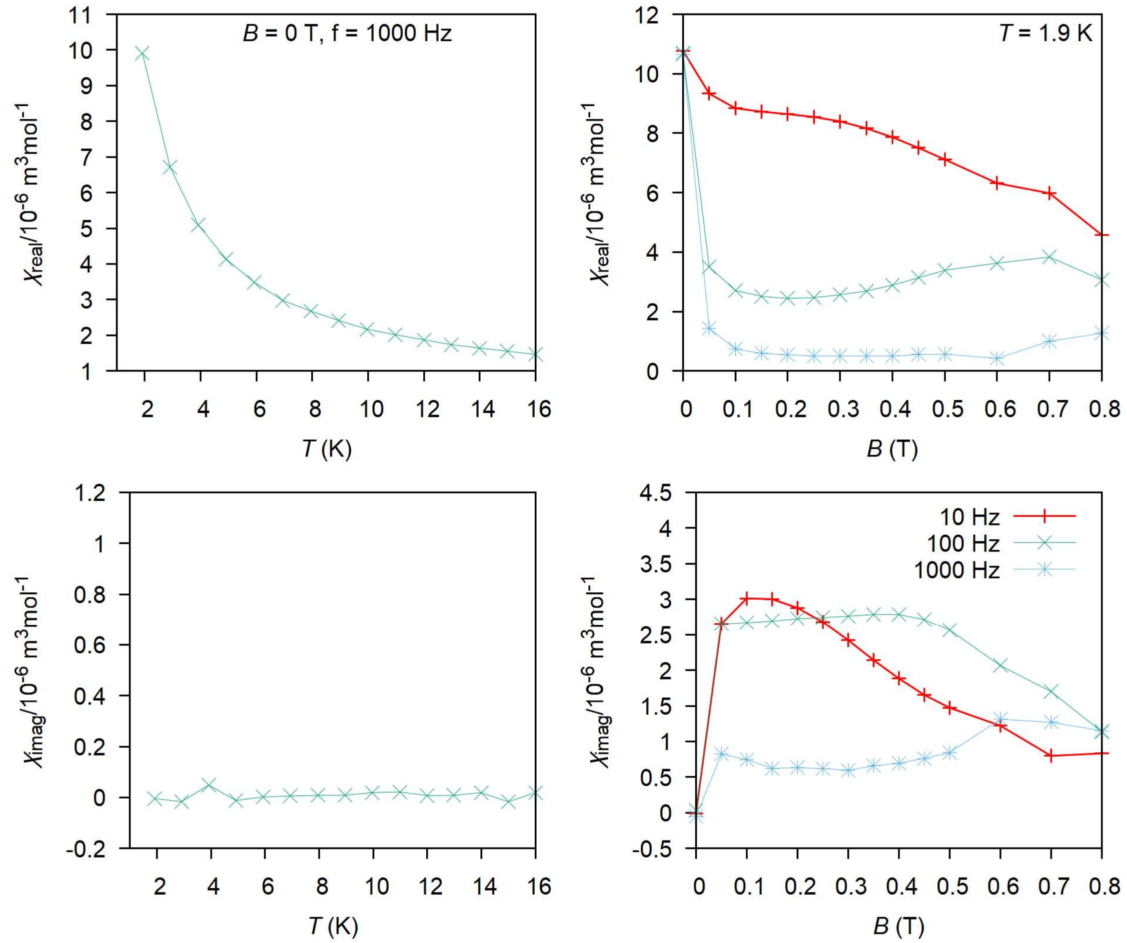

Figure S1. In-phase χ_{real} and out-of-phase χ_{imag} molar susceptibilities for **2** at zero static magnetic field (*left*) and in non-zero static field (*right*). Lines serve as guides for the eyes.
